# Granulocyte macrophage colony-stimulating factor receptor α expression and its targeting in antigen-induced arthritis and inflammation

**DOI:** 10.1186/s13075-016-1185-9

**Published:** 2016-12-01

**Authors:** Andrew D. Cook, Cynthia Louis, Matthew J. Robinson, Reem Saleh, Matthew A. Sleeman, John A. Hamilton

**Affiliations:** 1 Department of Medicine, Royal Melbourne Hospital, University of Melbourne, Parkville, Victoria 3050 Australia; 2 Department of Respiratory, Inflammation and Autoimmunity, MedImmune Ltd, Granta Park, Cambridge, CB21 6GH UK; 3 Present Address: Regeneron, 777 Old Saw Mill River Rd, Tarrytown, NY USA

**Keywords:** Granulocyte macrophage colony-stimulating factor, Arthritis, Inflammation, Targeting, Macrophages, Animal models

## Abstract

**Background:**

Blockade of granulocyte macrophage colony-stimulating factor (GM-CSF) and its receptor (GM-CSFRα) is being successfully tested in trials in rheumatoid arthritis (RA) with clinical results equivalent to those found with neutralization of the current therapeutic targets, TNF and IL-6. To explore further the role of GM-CSF as a pro-inflammatory cytokine, we examined the effect of anti-GM-CSFRα neutralization on myeloid cell populations in antigen-driven arthritis and inflammation models and also compared its effect with that of anti-TNF and anti-IL-6.

**Methods:**

Cell population changes upon neutralization by monoclonal antibodies (mAbs) in the antigen-induced arthritis (AIA) and antigen-induced peritonitis (AIP) models were monitored by flow cytometry and microarray. Adoptive transfer of monocytes into the AIP cavity was used to assess the GM-CSF dependence of the development of macrophages and monocyte-derived dendritic cells (Mo-DCs) at a site of inflammation.

**Results:**

Therapeutic administration of a neutralizing anti-GM-CSF mAb, but not of an anti-colony-stimulating factor (anti-CSF)-1 or an anti-CSF-1R mAb, ameliorated AIA disease. Using the anti-GM-CSFRα mAb, the relative surface expression of different inflammatory myeloid populations was found to be similar in the inflamed tissues in both the AIA and AIP models; however, the GM-CSFRα mAb, but not neutralizing anti-TNF and anti-IL-6 mAbs, preferentially depleted Mo-DCs from these sites. In addition, we were able to show that locally acting GM-CSF upregulated macrophage/Mo-DC numbers via GM-CSFR signalling in donor monocytes.

**Conclusions:**

Our findings suggest that GM-CSF blockade modulates inflammatory responses differently to TNF and IL-6 blockade and may provide additional insight into how targeting the GM-CSF/GM-CSFRα system is providing efficacy in RA.

**Electronic supplementary material:**

The online version of this article (doi:10.1186/s13075-016-1185-9) contains supplementary material, which is available to authorized users.

## Background

Clinical trials assessing blockade of granulocyte macrophage colony-stimulating factor (GM-CSF) or its receptor (GM-CSFRα) have commenced in rheumatoid arthritis (RA), psoriasis, multiple sclerosis and asthma, with some encouraging RA data [1, 2]. Questions, such as which is the key cell type(s) regulated by GM-CSF and whether it has pro-survival, differentiation and/or activation functions, remain to be addressed. For example, there is debate as to whether during an inflammatory response differentiation of inflammatory, monocyte-derived dendritic cells (Mo-DCs) is GM-CSF-dependent [3–9]. Given that anti-TNF and anti-IL-6 therapies have been successful in RA and that head-to-head trials between anti-GM-CSFRα and anti-TNF are ongoing [2], it would be useful to know how similar or not the biology of the pro-inflammatory activity of GM-CSF is to the respective biology of these other cytokines.

The basic unit structure of the dodecameric GM-CSF receptor (GM-CSFR) consists of a binding, cytokine-specific α subunit and a signaling β subunit [10]. It has been reported that there is a significant increase in the number of GM-CSFR α-subunit (GM-CSFRα) positive synovial macrophages in the RA synovium and that GM-CSFRα neutralization suppresses disease activity in the murine collagen-induced arthritis model [11]. It would seem that a GM-CSFRα monoclonal antibody (mAb) may be a useful tool to define GM-CSFRα expression on GM-CSF-responsive cells driving an inflammatory response and to be able to compare the efficacy with an anti-ligand therapeutic strategy.

The murine monoarticular antigen-induced arthritis (AIA) model is a widely used inflammatory arthritis model and is characterized by infiltration of neutrophils and mononuclear cells, synovitis (pannus formation) and erosion of cartilage and bone, thus replicating several features similar to those in RA [12–17]. An advantage of the AIA model lies in the exactly defined initiation of the arthritis, elicited by antigen injection into the knee joint cavity [18]. Using either gene-deficient mice or antibody neutralization strategies it has been found that both TNF and IL-6 contribute to at least some extent to AIA progression [19–24]. As we have identified suppression of AIA disease and pain in GM-CSF^-/-^ mice [25], this particular model may be useful for comparing the effects of its blockade on myeloid cell populations with that of TNF or IL-6.

The sterile peritoneal cavity is a convenient location to induce inflammation, to analyse inflammatory cell populations and to study the evolution of the inflammatory response on account of the easy access to the peritoneal exudate. We developed the antigen-induced peritonitis (AIP) model [26] because it has elements of both innate and acquired immunity and it follows a similar priming and challenge protocol with the same antigen as the AIA model. We therefore reasoned that it may represent a convenient surrogate model for this particular arthritis model in which to study changes in cell populations. We have shown that it also demonstrates GM-CSF dependence [27] and have begun to explore the mode of action of GM-CSF as a pro-inflammatory cytokine using this model [28].

We report here that GM-CSFRα blockade leads to myeloid population changes in AIA and AIP, which differ to those observed with TNF or IL-6 blockade. Additionally, we show that an anti-GM-CSFRα mAb can be used to directly monitor surface GM-CSFRα expression by flow cytometry and that its administration can lead to similar effects on myeloid cell populations as ligand neutralization at a site of inflammation, including preferential reduction in Mo-DCs.

## Methods

### Mice

C57BL/6 mice (both CD45.1 and CD45.2) were obtained from WEHI, Kew (Victoria, Australia). *Csf1r*-EGFP (MacGreen) mice [29], backcrossed onto the C57BL/6 background, are bred in our on-site animal facility at the University of Melbourne. Mice deficient in both βc and β_IL-3_ [30], referred to here as *Csf2rb* ^*-/-*^ *Csf2rb2* ^*-/-*^ mice, backcrossed onto the C57BL/6 background, were supplied by A. Lopez (Hanson Institute, Adelaide, Australia). Mice were fed standard rodent chow and water *ad libitum*. Mice of both sexes, aged 8–12 weeks, were used; experiments were approved by The University of Melbourne Animal Ethics Committee.

### Antigen-induced models

Antigen-induced arthritis (AIA) was induced as previously described using methylated BSA (mBSA) as antigen [25]. Briefly, mice were immunized with mBSA (Sigma-Aldrich, St Louis, MO, USA), emulsified in complete Freund’s adjuvant (CFA), intradermally in the base of the tail on day -7 and arthritis was induced 7 days later (day 0) by an intra-articular (i.a.) injection of mBSA into the right knee, the left knee being injected with PBS. Histological analysis was performed on the knee joints, which were scored separately (0–3) for cellular infiltration, cartilage damage and bone erosion (H&E stain), and proteoglycan loss (Safranin O/fast green stain) [25].

Antigen-induced peritonitis (AIP) was induced as previously described again using mBSA [28]. Briefly, mice were immunized intradermally with mBSA, emulsified in CFA, as described for the AIA model above; 14 days later, the primary immunization protocol was repeated as a boost. Seven days later, mice were injected intraperitoneally (i.p.) with 200 μg mBSA to induce peritonitis (day 0).

### mAb treatment

Mice were treated i.p. with 150 μg anti-GM-CSF (22E9.11, J. Abrams) [28], 250 μg anti-CSF-1R (ASF98, S-I Nishikawa) [28], 150 μg anti-CSF-1 (F. Dodeller, MorphoSys, Munich, Germany) [28, 31], 750 μg anti-GM-CSFRα (CAM-3003) [11], 750 μg anti-TNF (MP6-XT22, Biolegend, San Diego, CA, USA), 750 μg anti-IL-6 (Biolegend) and their respective isotype control mAb, at the time points indicated.

### Cell isolation and fluorescence-activated cell sorting (FACS) analysis

Cell suspensions were prepared from the synovium or peritoneal cavity and analysed by flow cytometry [28, 32, 33]. For synovial cells, mice were perfused with 20 ml PBS and the patellae from the knee joints were dissected and the synovium digested (1 mg/ml collagenase type IV, 0.5 mg/ml neutral protease, 50 μg/ml DNase I in PBS) for 45 minutes at 37 ^o^C, then passed through a 70-μm nylon mesh to obtain a single cell suspension. Cells were washed twice in PBS, followed by cell counting using BD Trucount tubes (BD Biosciences). Joint cells were incubated with Fc block (anti-CD16/32, clone 2.4G2) and stained using the following antibodies: APC-Cy7-conjugated CD45 (30-F11), BV421-conjugated CD11b (M1/70), PE-Cy7-conjugated CD11c (N418), BV510-conjugated I-A/I-E (M5/114.15.2), FITC-conjugated Gr-1 (RB6-8C5), PE-conjugated F4/80 (BM8) and APC-conjugated GM-CSFRα (CAM-3003). Note that Gr-1 was used for staining synovial cells rather than Ly6G and Ly6C due to the number of available channels.

Peritoneal cells were collected by lavage with 5 ml cold PBS, followed by washing in PBS and cell counting using either trypan blue or BD Trucount tubes (BD Biosciences). Cells were incubated with Fc block (anti-CD16/32, clone 2.4G2) and stained using the following antibodies: PE-conjugated CD115 (AFS98), PE-TxRed or PE-Cy7-conjugated CD11b (M1/70), BV421-conjugated CD11c (HL3), BV510-conjugated I-A/I-E (M5/114.15.2), APC-Cy7 conjugated Ly6G (1A8), FITC- or PE-Cy7-conjugated Ly6C (HK1.4), FITC-conjugated CD45.1 (A20), FITC-conjugated CD45.2 (104) and APC-conjugated GM-CSFRα (CAM-3003).

All fluorochrome-conjugated antibodies were sourced from BD Biosciences, Biolegend, or eBioscience, with the exception of αGM-CSFRα (CAM-3003) mAb. CAM-3003 was conjugated with APC using a Lightning-Link antibody labelling kit (Innova Biosciences) according to manufacturer’s protocol. Cell viability was determined using 7-AAD (BD Biosciences) and data were acquired on a CyAn flow cytometer (Beckman Coulter). Compensation was acquired using single-stained samples and specificity of antibody staining was determined by the fluorescence-minus-one method. Analysis was performed using Kaluza 1.2 software (Beckman Coulter).

### Adoptive cell transfer

Bone marrow was flushed from the tibias and femurs of donor mice, red blood cells lysed and CD115^+^ cells were either MACS-enriched, using CD115-Biotin antibody and anti-Biotin microbeads (Miltentyi Biotec), or FACS sorted. Monocyte purity after enrichment was >90%; 1.0 × 10^6^ enriched monocytes were transferred i.p. into mBSA-challenged AIP mice on day 2.

### Gene expression analysis

Total RNA was isolated (Qiagen) from total peritoneal exudate cells (PECs), magnetic bead-isolated CD115^+^ PECs, or sorted CD115^+^ CD45.1^+^ donor PECs, from day 4 AIP. Individual gene expression was measured by RT-PCR using TaqMan Gene Expression Arrays (ThermoFisher). For transcriptomic analysis, RNA was quantified, normalised and verified by Bioanalyzer (Agilent) prior to processing onto Genechip Mouse Gene 2.0ST Microarrays (Affymetrix). Sorted CD115^+^ donor PECs additionally underwent PCR amplification prior to analysis (Nugen). Normalisation across all arrays was achieved using the robust multi-array average (RMA) expression measure [34] which results in expression measures (summarised intensities) in log base 2. Significant genes from each comparison were analysed for enrichment of Kyoto Encyclopedia of Genes and Genomes (KEGG) pathway membership using a hypergeometric test. Pathway enrichment (*p* < 0.05) was assessed separately for upregulated and downregulated genes.

### Statistical analysis

Data are expressed as mean ± SEM. Statistical differences were assessed using the unpaired Student’s *t* test or one-way analysis of variance (ANOVA). For histologic scores, Kruskal-Wallis one-way ANOVA was used. *P* ≤0.05 was considered statistically significant. In the microarray analysis, differentially expressed genes were defined as fold change ≥2 with an adjusted *p* value <0.01. Empirical Bayesian analysis was applied (including vertical within a given comparison) and the *p* value was adjusted for multiple testing.

## Results

### GM-CSF, but not CSF-1, neutralization suppresses AIA

**Fig. 1:**
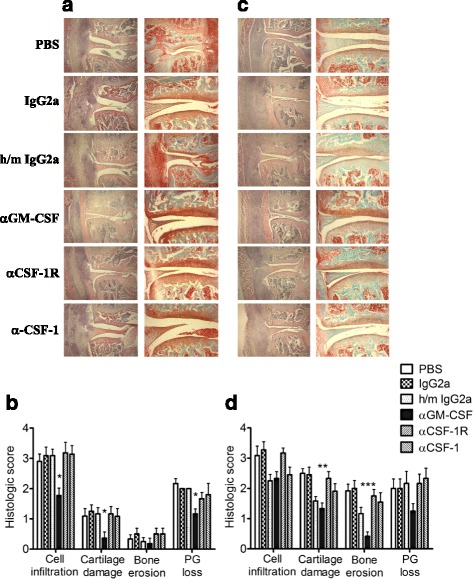
Granulocyte macrophage colony-stimulating factor (*GM-CSF*) but not colony-stimulating factor (*CSF-1*) neutralization suppresses antigen-induced arthritis (AIA). AIA-primed mice were treated with PBS, anti-GM-CSF, anti-CSF-1R, anti-CSF-1 or isotype monoclonal antibodies, either prophylactically (days -2 and 0) (**a** and **b**) or therapeutically (days 2 and 4) **(c** and **d**), with arthritis being induced on day 0 by intra-articular injection of methylated bovine serum albumin (mBSA). Histological analysis of the arthritic joints was performed on day 3 (**a** and **b**) and day 7 (**c** and **d**) post arthritis induction. **a**, **c** Representative H&E (*left*) and Safranin O/fast green (*right*) stained sections. **b**, **d** Quantification of histological appearances. *PG* proteoglycan. Data are expressed as mean + SEM, n = 6–12 mice/group; *p < 0.05, **p < 0.01, ***p < 0.001, anti-GM-CSF vs. PBS or IgG2a

We have previously shown, using knockout mice, that AIA is partially dependent on GM-CSF [25]; however, this approach cannot delineate whether GM-CSF is acting during the antigen-priming, antigen-challenge (effector) and/or the more chronic inflammatory phase. Therefore to explore when GM-CSF might be acting in relation to AIA disease induction, we studied the effectiveness of both prophylactic and therapeutic treatment with a neutralizing anti-GM-CSF mAb (22E9). Treating AIA-primed mice prophylactically with anti-GM-CSF mAb, on days -2 and 0, led to some reduction in cell infiltration 3 days after AIA induction (day 0), as judged by histological analysis (H&E stain) compared to isotype mAb treatment (Fig. 1a and b). There was also significantly less cartilage damage (H&E stain) and proteoglycan loss (Safranin O/fast green stain) in the former group (Fig. 1a and b). Treating AIA-primed mice therapeutically on days 2 and 4 post AIA induction (day 0) led to a trend towards a reduction in cell infiltration and proteoglycan loss at day 7 and a significant reduction in the degree of cartilage damage and bone erosion compared to isotype-treated and PBS-treated AIA-primed mice (Fig. 1c and d). Treating mice with anti-GM-CSF mAb on days 9 and 11 post AIA onset (chronic phase) had no effect on cell infiltration but did lead to a significant reduction in bone erosion and a trend towards a reduction in cartilage damage at day 14 compared to isotype-treated mice (data not shown). Thus, the earlier the anti-GM-CSF mAb treatment was started the more significant was the reduction in cell infiltration, while both prophylatic and therapeutic anti-GM-CSF mAb treatments resulted in reduced joint damage, i.e., GM-CSF blockade during either the acute or the more chronic phase of AIA ameliorated the structural changes.

We also assessed whether CSF-1, which acts more specifically on populations of the mononuclear phagocyte system (MPS) via CD115 (c-Fms) [35], is involved in AIA progression. AIA-primed mice were treated both prophylactically and therapeutically with an anti-CSF-1 mAb; however, it had no effect on cell infiltration or joint damage (Fig. 1a-d). As there is another ligand (IL-34) for the CSF-1R [1], we also tested an anti-CSF-1R mAb and obtained the same negative findings, suggesting that neither CSF-1 nor IL-34 is critical for AIA progression.

### GM-CSFRα expression and the effect of its blockade on myeloid cell populations in AIA

It has been previously shown that in the synovial tissue of patients with RA there is a significant increase in GM-CSFRα-expressing cells and that the receptor is expressed by macrophages [11]. GM-CSFRα neutralization using the mAb, CAM-3003, was shown to be as effective as anti-GM-CSF and anti-TNF mAbs in suppressing manifestations of murine collagen-induced arthritis, including the degree of synovial inflammation [11, 33, 36, 37]; the clinical benefit is also similar in RA trials using GM-CSF receptor or GM-CSF neutralizing mAbs [2, 38]. For these and subsequent experiments we utilized the GM-CSFRα blocking mAb, CAM-3003, both as a neutralizing antibody in vivo and to detect GM-CSFRα expression by flow cytometry. This allowed us to study the expression of the receptor on specific cell populations at sites of inflammation and to verify that our experiments were not compromised by a lack of target coverage in the anti-GM-CSFRα-treated mice, by assessing receptor occupancy simultaneously with the changes in their respective cell numbers.

**Fig. 2:**
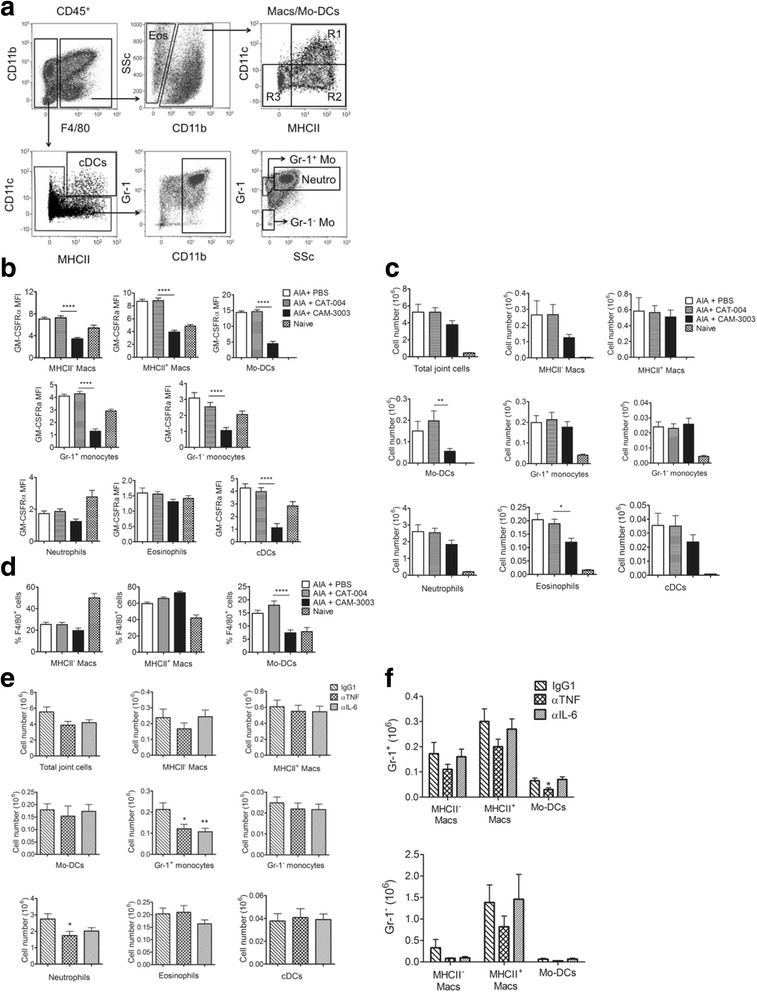
Effect of granulocyte macrophage colony-stimulating factor receptor α (*GM-CSFRα*), TNF and IL-6 blockade on myeloid cell populations in antigen-induced arthritis (*AIA*). **a** Representative FACS plots showing the gating strategy used to identify CD45^+^ myeloid populations in the AIA knee joint. F4/80^int^SSc^hi^ eosinophils (*Eos*), F4/80^+^CD11c^+^MHCII^+^ monocyte-derived dendritic cells (*Mo-DCs*) (*R1*), F4/80^+^CD11c^-^MHCII^+^ macrophages (*Macs*) (*R2*), F4/80^+^CD11c^-^MHCII^-^ macrophages (*R3*), F4/80^-^CD11c^+^ MHCII^+^ conventional dendritic cells (*cDCs*), F4/80^-^CD11b^+^SSc^int^ Gr-1^+^ neutrophils (*Neutro*) and F4/80^-^CD11b^+^SSc^lo^Gr-1^+/-^ monocytes (*Mo*). **b**-**d** AIA-primed mice were treated with PBS, CAT-004 isotype monoclonal antibody (mAb) or CAM-3003 mAb on day -1 and day 2. Cells were harvested at day 5 post AIA induction and synovial populations analysed. **b** GM-CSFRα expression on myeloid cell populations. **c** Total CD45^+^ cells and myeloid cell populations. **d** MHCII^-^ macrophages, MHCII^+^ macrophages and Mo-DCs as a percentage of F4/80^+^ cells. **e**-**f** AIA-primed mice were treated with anti-TNF mAb, anti-IL-6 mAb or IgG1 isotype mAb on day -1 and day 2. Cells were harvested at day 5 post AIA induction and synovial populations analysed. **e** Total cells and myeloid cell populations. **f** Number of Gr-1^+^ and Gr-1^-^ cells in the different F4/80^+^ mononuclear phagocyte system (MPS) cell populations. Data are expressed as mean ± SEM; n = 10–16 mice/group; *p < 0.05, **p < 0.01, ***p < 0.001, ****p < 0.0001, CAT-004 vs. CAM-3003, or anti-TNF or anti-IL-6 vs. IgG1

Notwithstanding the challenges faced in defining categorically mononuclear phagocyte system (MPS) populations [28, 39–42], we first identified the different synovial myeloid populations, using a similar gating strategy to the one we previously published for the AIP model [28], with some notable modifications - the marker F4/80 was used in place of CD115 (CSF-1R) for analysis of the synovial macrophage/Mo-DC populations, as done by Weiss et al. [17], because surface CD115 could not be detected following tissue digestion and because we have previously shown that the CD115^+^ populations in the inflamed AIP peritoneal cavity are also F4/80^+^ [28]. The gating strategy is provided in Fig. 2a using the following markers: CD45^+^ F4/80^-^CD11b^+^ SSc^int^ Gr-1^+^ neutrophils, CD45^+^F4/80^+^CD11b^+^ MPS populations (CD11c^+^MHCII^+^ Mo-DCs (R1), CD11c^-^MHCII^+^ macrophages (R2), CD11c^-^MHCII^-^ macrophages (R3)), CD45^+^F4/80^-^CD11b^+^SSc^lo^Gr-1^+/-^ monocytes, CD45^+^F4/80^-^CD11c^+^ MHCII^+^ conventional dendritic cells (cDCs) and CD45^+^F4/80^int^SSc^hi^ eosinophils. Anti-Gr-1 mAb stains both Ly6G and Ly6C. We confirmed that the neutrophils were Ly6G^+^Ly6C^+^, while the F4/80^-^CD11b^+^SSc^lo^ monocytes were Ly6G^-^ and either Ly6C^+^ or Ly6C^-^ (Additional file 1A). Furthermore, we showed that Ly6G^+^ neutrophils were CD64^-^ and F4/80^+^ macrophages/Mo-DCs were CD64^+^ (Additional file 1B).

Following AIA induction there was an influx of CD45^+^ cells into the joint, with neutrophils being the predominant cell type, comprising 50.5 ± 1.6% of CD45^+^ synovial cells at day 5. F4/80^+^ MPS populations (comprising Mo-DCs (R1) and MHCII^+/-^ macrophages (R2 and R3, respectively) (Fig. 2a)) made up a further 17.6 ± 2.0% of CD45^+^ synovial cells, with monocytes (4.8 ± 0.7%), eosinophils (4.7 ± 0.4%) and cDCs (0.6 ± 0.1%) being minor populations. Using the CAM-3003 mAb, GM-CSFRα was highly expressed on each of the F4/80^+^ MPS populations, monocytes and cDCs (Fig. 2b) and further enhanced during inflammation, whereas on neutrophils and eosinophils its levels were low (Fig. 2b) and barely detectable above that of the isotype control (data not shown). AIA-primed mice were treated with the mAbs on days -1 and 2 (with i.a. challenge with mBSA again at day 0), and synovial cells were analysed once more on day 5. This prophylactic treatment protocol was chosen as it covers the time period when cell infiltration into the joint is maximal, thus, allowing us to determine the effect of mAb blockade on the infiltrating cell populations. CAM-3003 treatment, but not that with the isotype control, CAT-004, reduced the levels of detectable GM-CSFRα on the F4/80^+^ MPS populations, monocytes and cDCs (Fig. 2b), suggesting that the availability of free receptor was reduced on these cell types. The low receptor levels on neutrophils and eosinophils meant that a similar analysis could not be conducted.

As regards changes in cell population numbers following prophylactic CAM-3003 treatment (days -1 and 2), there were significantly fewer Mo-DCs and eosinophils (vs. CAT-004 isotype control) in AIA mice at day 5 (Fig. 2c); there was also a trend towards fewer neutrophils, CD11c^-^MHCII^-^ macrophages and cDCs, but not towards fewer CD11c^-^MHCII^+^ macrophages or Gr-1^+/-^ monocytes. Interestingly, as a proportion of F4/80^+^ cells, Mo-DCs were the only cell type to be significantly lower following CAM-3003 treatment indicating a preferential reduction (Fig. 2d). Gr-1 expression varied on the different F4/80^+^ subpopulations, with 66.6 ± 4.3% of CD11c^-^MHCII^-^ macrophages, 42.4 ± 3.5% CD11c^-^MHCII^+^ macrophages and 25.6 ± 3.4% Mo-DCs being Gr-1^+^ at day 5. CAM-3003 treatment had no effect on the proportion of Gr-1^+^ cells in each MPS population (69.0 ± 3.0% CD11c^-^MHCII^-^ macrophages, 45.2 ± 2.8% CD11c^-^MHCII^+^ macrophages and 34.7 ± 3.1% Mo-DCs) (data not shown).

TNF and IL-6 have been implicated in AIA pathogenesis [19–24], and blocking antibodies against them have therapeutic effects in RA [43]. We reasoned that mechanistic comparisons between GM-CSFRα neutralization and that of these other pro-inflammatory cytokines may be informative. We therefore explored whether TNF or IL-6 blockade led to similar changes in AIA cell populations as GM-CSFRα blockade. As for the CAM-3003 protocol above, mice undergoing AIA were treated both before (day -1) and after (day 2) i.a. antigen challenge with anti-TNF or anti-IL-6 mAbs and their isotype control, with mice being killed at day 5. There was a trend for fewer total cells and neutrophils (vs. IgG1 isotype control) in both anti-TNF-treated and anti-IL-6 treated AIA mice, being statistically significant for the latter cell type for anti-TNF mAb treatment (Fig. 2e), thus confirming previous findings [24, 44]. However, unlike CAM-3003-treated mice (Fig. 2c), no such significant decreases in cell numbers were noted for Mo-DCs or eosinophils following anti-TNF or anti-IL-6 mAb treatment (Fig. 2e).

There were also fewer synovial Gr-1^+^ monocytes following anti-TNF mAb (Fig. 2e). In line with this, following anti-TNF treatment, significantly fewer Mo-DCs were Gr-1^+^ and there was a trend towards fewer MHCII^-^ and MHCII^+^ macrophages being Gr-1^+^ (Fig. 2f). The reduction in synovial Ly6C^+^ monocyte number also occurred in anti-IL-6-treated mice (Fig. 2e); however, this treatment did not affect the numbers of Gr-1^+^ F4/80^+^ MPS populations (Fig. 2f). There were no differences in the numbers of Gr-1^-^ MPS cells between any of the treatment groups (Fig. 2e and f).

Thus, in AIA GM-CSFRα was most highly expressed on MPS cells and cDCs in the synovium; also administration of a neutralizing GM-CSFRα mAb variably reduced the synovial myeloid populations with a disproportionate effect on Mo-DCs amongst MPS populations. TNF and IL-6 neutralization had no significant effect on the numbers of synovial Mo-DCs and eosinophils, but reduced the numbers of synovial Gr-1^+^ monocytes.

### GM-CSFRα expression and the effect of its prophylactic blockade in AIP

To characterize the effect of GM-CSFRα blockade on myeloid cells in greater detail we utilized our AIP model [28]. It should be noted that the protocol for induction of AIP is quite similar to AIA, except for the site of antigen (mBSA) challenge [25–28].

PEC populations were again analysed by flow cytometry. We again used CD115 [28, 32], when analysing PECs, given its specificity for macrophage-lineage cells amongst haemopoietic populations. The gating strategy was as before [28] (Additional file 2) using the following markers: Ly6G^+^ neutrophils, Ly6G^-^CD115^+^ monocytes/macrophages/Mo-DCs (CD11c^+^MHCII^+^ Mo-DCs (R1), CD11c^-^MHCII^+^ macrophages (R2), CD11c^-^MHCII^-^ monocytes (R3)), CD115^-^Ly6G^-^CD11c^+^MHCII^+^ cDCs and CD115^-^Ly6G^-^CD11b^int^SSc^hi^ eosinophils. Note that, as previously [28], the CD115^+^CD11c^-^MHCII^-^ cells (R3) in the inflamed peritoneal cavity are referred to as monocytes, based on their morphology and their being predominantly Ly6C^+^.

**Fig. 3:**
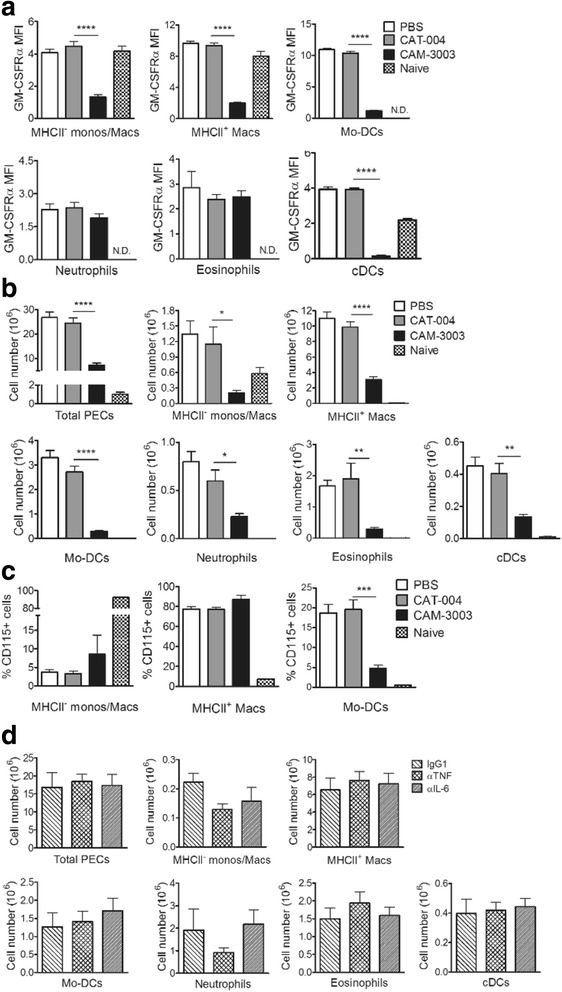
Effect of granulocyte macrophage colony-stimulating factor receptor α (*GM-CSFRα*), TNF and IL-6 blockade on myeloid cell populations in antigen-induced peritonitis (AIP). **a**-**c** AIP-primed mice were treated with PBS, CAT-004 isotype monoclonal antibody (mAb) or CAM-3003 mAb on day -1. Cells were harvested at day 4 post AIP induction and peritoneal exudate cell (*PEC*) populations analysed. **a** GM-CSFRα expression on myeloid cell populations. **b** Total cells and myeloid cell populations. **c** MHCII^-^ monocytes/macrophages, MHCII^+^ macrophages and monocyte-derived dendritic cells (*Mo-DCs*) as a percentage of CD115^+^ cells. **d** AIP-primed mice were treated with anti-TNF mAb, anti-IL-6 mAb or IgG1 isotype mAb on day -1 and day 2. Cells were harvested at day 4 post AIP induction and PEC populations analysed. Total cells and myeloid cell populations are shown. Data are expressed as mean ± SEM; n = 8 mice/group; *p < 0.05, **p < 0.01, ***p < 0.001, ****p < 0.0001 for CAT-004 vs. CAM-3003. *cDCs* conventional dendritic cells

Following AIP induction, in PBS-treated mice at day 4 (the peak of the cellular response [26, 28]) there was an influx of cells with 15.8 ± 2.8% of exudate cells being neutrophils, 47.6 ± 1.9% CD115^+^ cells (monocytes, macrophages, Mo-DCs), 6.7 ± 0.6% eosinophils and 2.6 ± 0.2% cDCs. As for the synovial cells in the AIA model, the CAM-3003 mAb was able to detect GM-CSFRα on the MPS cells (CD115^+^) and on cDCs (Fig. 3a), whereas again detection on neutrophils and eosinophils was difficult (low mean fluorescence intensity (MFI)) (Fig. 3a) and was barely above that of the isotype control (data not shown). Similar to what was shown for the AIA model (Fig. 2b), in the AIP model CAM-3003 treatment at day -1, but not that with CAT-004, reduced the levels of detectable GM-CSFRα on the CD115^+^ cells and cDCs (Fig. 3a).

Upon prophylactic CAM-3003 administration (day -1), at day 4 there were also significantly fewer total PECs, CD11c^-^MHCII^-^ monocytes, CD11c^-^MHCII^+^ macrophages, Mo-DCs, neutrophils, eosinophils and cDCs with 30 mg/kg CAM3003 given i.p. (Fig. 3b), a dose found to be maximal - interestingly, the effect in general on cell population numbers was more pronounced than in the AIA model with data for the CD11c^-^MHCII^+^ peritoneal macrophages differing from the lack of effect of CAM-3003 shown above for the CD11c^-^MHCII^+^ synovial macrophages (Fig. 2c). As a proportion of CD115^+^ cells, only the Mo-DCs were reduced following CAM-3003 treatment once again indicating their preferential reduction (Fig. 3c), similar to that previously reported for the AIP model following blockade of GM-CSF [28] and to the data above for the AIA model (Fig. 2d). CAM-3003 treatment had no effect on the proportion of Ly6C^+^ cells within each CD115^+^ subpopulation (data not shown). Interestingly, the proportion of MHCII^+^ macrophages and Mo-DCs in AIP which were Ly6C^+^ was significantly lower than in AIA (MHCII^+^ macrophages: 21.7 ± 2.3% vs. 38.6 ± 3.1%, Mo-DCs 4.0 ± 0.4% vs. 24.4 *±* 1.5%, AIP vs. AIA).

We next explored whether or not TNF or IL-6 blockade led to similar changes in AIP cell populations as GM-CSFRα blockade using a similar administration protocol. Mice undergoing AIP were treated before (day -1) i.p. antigen challenge, i.e., prophylactically, with anti-TNF, anti-IL-6 mAbs or their isotype control, with mice killed at day 4; exudate cell populations were again analysed by flow cytometry. Unlike anti-GM-CSFRα (Fig. 3a), neither anti-TNF nor anti-IL-6 treatment had significant effects on myeloid population numbers (Fig. 3d), nor on detectable GM-CSFRα levels (data not shown), although there was a trend for fewer MHCII^-^ monocytes/macrophages in anti-TNF-treated mice (Fig. 3d).

Thus, as in AIA, GM-CSFRα in AIP can be detected on certain myeloid populations and anti-GM-CSFRα administration led to a preferential reduction of Mo-DCs, indicating again the convenience of the AIP model in understanding the role of GM-CSF in inflammation. TNF and IL-6 neutralization had no significant effect on the numbers of different PEC populations compared to GM-CSFRα neutralization.

### Gene expression in PECs following GM-CSFRα blockade in the AIP model

We repeated the above experiment with CAM-3003 and performed microarray analysis on purified CD115^+^ peritoneal cells (day 4) from the AIP cavity. As the majority of these cells would have derived from circulating monocytes [28], we first compared their gene expression to monocytes. Over 2000 genes were significantly different between these two populations (>2 fold change in expression and adjusted *p* value <0.01). However, only 12 genes were significantly changed between isotype-treated and CAM-3003 treated mice with this particular degree of stringency (Additional file 3). Although no genes were changed between isotype- and PBS-treated AIP mice, an additional 36 genes were found to be differentially expressed between the PBS and CAM-3003 groups (Additional file 3). Consequently, we performed pathway analysis on all the genes that differed between CAM-3003-treated mice and the PBS-treated or isotype-treated mice. Using KEGG pathway enrichment analysis, the only pathways that were significantly changed were driven by one or two genes. Therefore GM-CSFRα blockade can by and large reduce the number of infiltrating myeloid cell populations, but had, at least at the day 4 time point in this model, a surprisingly minor impact on the CD115^+^ cell transcriptome (see “Discussion”).

### Therapeutic blockade with anti-GM-CSFRα mAb in the AIP model

**Fig. 4:**
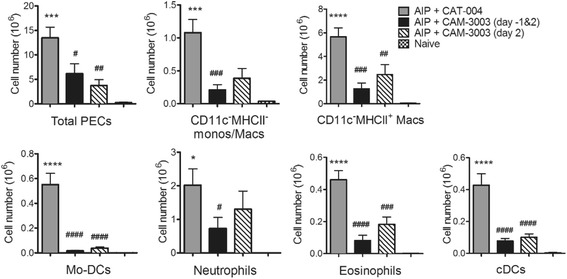
Therapeutic blockade with anti-granulocyte macrophage colony-stimulating factor receptor α (anti-GM-CSFRα) monoclonal antibody (mAb) in antigen-induced peritonitis (AIP). Peritoneal exudate cells (*PECs*) were harvested from naïve mice or day 4 after AIP induction from mice treated with CAT-004 (day -1 and day 2), CAM-3003 (day -1 and day 2) and CAM-3003 (day 2) and PEC populations were analysed (FACS). Data are expressed as mean ± SEM; *n* = 8 mice/group; *p < 0.05, ***p < 0.001, ****p < 0.0001, vs. naïve; ^#^ *p* < 0.05, ^##^ *p* < 0.01, ^###^ *p* < 0.001, ^####^ *p* < 0.0001, vs CAT-004. *Mo-DCs* monocyte-derived dendritic cells, *cDCs* conventional dendritic cells

In order to gain insight as to when GM-CSF signalling is required during an inflammatory reaction we next explored whether in the AIP model a therapeutic delivery (at day 2) of CAM-3003 after antigen challenge was effective and, if so, was it in anyway more effective if an additional prophylactic delivery (at day -1) was given, the latter protocol being similar to that employed above in the AIA model (Fig. 2). It can be seen that at day 4 the reductions in total PEC numbers and those of the various myeloid populations upon therapeutic treatment with CAM-3003 were similar to those noted if a pretreatment was incorporated in the protocol (Fig. 4), although a single treatment at day 2 did not lead to a significant reduction in neutrophils, in keeping with them being the predominant cell type early in an inflammatory response. Once again, detectable surface GM-CSFRα levels were significantly lower on CD115^+^ cells and cDCs following both CAM-3003 treatment protocols (data not shown).

### Anti-GM-CSFRα mAb suppresses development of Mo-DCs from donor monocytes in the AIP cavity

**Fig. 5:**
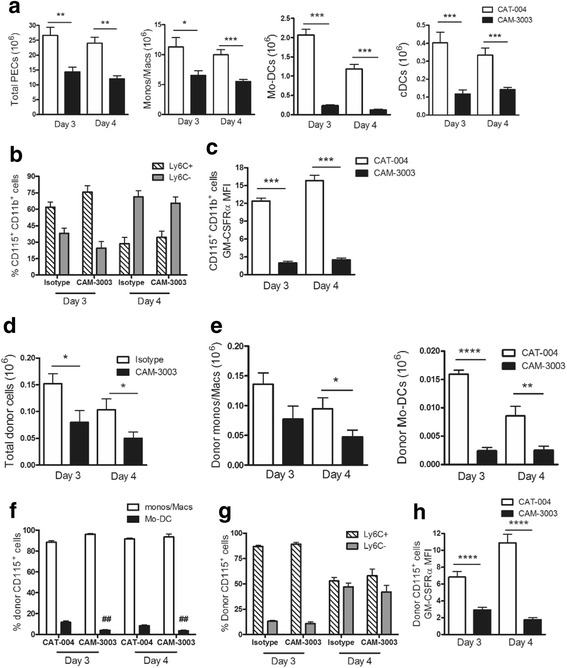
Granulocyte macrophage colony-stimulating factor receptor α (GM-CSFRα) blockade suppresses generation of monocyte-derived dendritic cells (Mo-DCs) from donor monocytes in the antigen-induced peritonitis (AIP) cavity. CD115^+^ MacGreen bone marrow cells were adoptively transferred intraperitoneally (i.p.) to C57BL/6 wild-type (WT) mice on day 2 following AIP induction. C57BL/6 mice were treated i.p. with CAT-004 or CAM-3003 at the same time as the CD115^+^ cell transfer. Peritoneal exudate cells (PECs) were harvested on day 3 and day 4 post AIP induction, i.e., one or two days, respectively, following CD115^+^ cell transfer and monoclonal antibody (mAb) treatment. **a** Total cells, monocytes/macrophages (CD11c^-^MHCII^+/-^), Mo-DCs and conventional dendritic cells (cDCs) (host and donor cells). **b** Percentage of CD115^+^CD11b^+^ cells that are Ly6C^+^ vs. Ly6C^-^ (host and donor cells). **c** GM-CSFRα expression (mean fluorescence intensity (MFI)) on CD115^+^CD11b^+^ cells (host and donor cells). **d** Total numbers of donor cells recovered. **e** Numbers of donor monocytes/macrophages (CD11c^-^MHCII^+/-^) and Mo-DCs recovered. **f** Percentage of recovered donor cells that are monocytes/macrophages (CD11c^-^MHCII^+/-^) and Mo-DCs. **g** Percentage of recovered donor cells that are Ly6C^+^ vs. Ly6C^-^. **h** GM-CSFRα expression (MFI) on recovered donor cells. Data are expressed as mean ± SEM; *n* = 8 mice/group; *p < 0.05, **p < 0.01, ***p < 0.001, ****p < 0.0001, CAM-3003 vs. CAT-004; ^##^ *p* < 0.01, CAM-3003 vs. CAT-004

The above data in the AIA and AIP models suggests that GM-CSF signalling is most profoundly controlling the generation of Mo-DCs from monocyte precursors. To determine whether this GM-CSF-dependent phenotypic change was actually occurring in recruited monocytes in the inflamed peritoneal cavity we injected MacGreen (*Csf1R-*EGFP) bone marrow monocytes [28] i.p. at day 2 into C57BL/6 wild-type (WT) recipient mice undergoing AIP, in the presence or absence of CAM-3003 - we previously used this monocyte donor approach to demonstrate the monocyte origin of the AIP CD115^+^ exudate populations including Mo-DCs [28]. As above at day 4, as well as at day 3, there were fewer total PECs, monocytes/macrophages (R2 and R3 (MHC^+/-^ populations combined), Mo-DCs and cDCs if CAM-3003 was administered (Fig. 5a). Also, the percentage of CD115^+^ cells that expressed Ly6C^+^ declined over time but this decline was not altered by CAM-3003 (Fig. 5b). Again, detectable GM-CSFRα surface expression on the total CD115^+^ PECs was reduced by CAM-3003 (Fig. 5c).

For the total donor (GFP^+^) cells, fewer were retrieved from the cavity of CAM-3003-treated mice compared to CAT-004 isotype-treated mice (Fig. 5d). We could not detect donor cells in the draining lymph nodes consistent with there being a pro-survival effect of GM-CSF rather than an influence on cell migration (data not shown). There were significantly fewer donor Mo-DCs at days 3 and 4 in the CAM-3003-treated mice and a trend towards fewer donor monocytes/macrophages (R2 and R3 populations combined) on day 3, which reached significance on day 4 (Fig. 5e). Again the proportion of CD115^+^ donor cells converting into Mo-DCs was significantly reduced by CAM-3003 treatment (Fig. 5f); however, the loss of surface Ly6C was not affected (Fig. 5g). Interestingly, there appeared to be an increase over time in detectable surface GM-CSFRα in the donor monocytes (CAT-004-treated group, Fig. 5h).

Therefore, following transfer of monocytes directly into the inflamed peritoneum in the presence of CAM-3003, the results suggest that the observed broad regulation in MPS and cDC numbers and the disproportionate impact on Mo-DCs can be driven by GM-CSF in the inflammatory milieu, although some additional impact on monocyte recruitment cannot be discounted.

### Transcriptome changes in donor CD115^+^ monocyte-derived populations following GM-CSFRα blockade in the AIP model

We repeated the above monocyte transfer experiment using CD115^+^ CD45.1 congenic monocytes transferred into a CD45.2 host with very similar results (data not shown) - the donor-derived cells were then isolated and subjected to microarray analysis so that we could examine the effect of GM-CSF on the transcriptome of this population. Fewer genes were significantly changed compared with the experimental design shown in Additional file 3, with 889 genes changing between the transferred monocytes and those harvested after 2 days (CD45.1^+^). CAM-3003 administration affected few genes with only three being changed compared to the CAT-004 isotype control and an additional 20 genes relative to the PBS control (Additional file 4). *Retnla* (Fizz1) and *Ear1* were decreased and *Cd36* increased in CAM-3003-treated vs. CAT-treated cells. Any KEGG pathway enrichment was again driven by single gene changes. With this approach in the AIP model, GM-CSF, at least at the time point examined, once more made only a minor contribution to the gene expression changes in monocyte-derived cells (but see “Discussion”). Decreased gene expression of *Retnla* and increased gene expression of *Cd36* following GM-CSFRα blockade in total PECs from a day 4 AIP model was confirmed by quantitative PCR (data not shown).

### GM-CSFR signalling in monocytes regulates Mo-DC/macrophage numbers in the AIP model

**Fig. 6:**
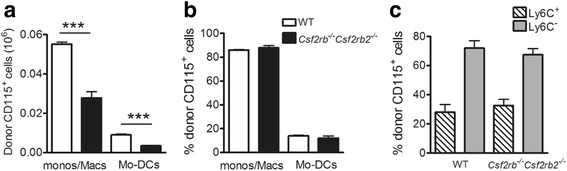
In antigen-induced peritonitis (AIP), granulocyte macrophage colony-stimulating factor receptor (GM-CSFR) signalling in monocytes directly regulates monocyte/macrophage/monocyte-derived dendritic cell (*Mo-DC*) numbers but not Mo-DC development. CD115^+^ bone marrow monocytes from *Csf2rb* ^*-/-*^ *Csf2rb2* ^*-/-*^ or C57BL/6 wild-type (*WT*) mice (both CD45.2) were adoptively transferred intraperitoneally (i.p.) to C57BL/6 (CD45.1) mice on day 2 following AIP induction. Peritoneal exudate cells (PECs) were harvested on day 4, i.e., two days following CD115^+^ cell transfer. **a** Numbers of donor monocytes/macrophages and Mo-DCs. **b** Percentage of donor cells that are monocytes/macrophages and Mo-DCs. **c** Percentage of donor cells that are Ly6C^+^ vs. Ly6C^-^. Data are expressed as mean ± SEM; n = 5–7 mice/group; ***p < 0.001, WT vs. *Csf2rb* ^*-/-*^ *Csf2rb2* ^*-/-*^ donor cells

In order to test whether GM-CSF in the AIP peritoneum was acting directly via its receptor on the incoming monocytes, *Csf2rb* ^*-/-*^ *Csf2rb2* ^*-/-*^ (CD45.2) donor monocytes (CD115^+^) were injected i.p. at day 2 into C57BL/6 WT (CD45.1) mice undergoing AIP. As can be seen in Fig. 6a, at day 4 there were fewer donor monocytes/macrophages (R2 and R3 populations combined) and Mo-DCs if *Csf2rb* ^*-*/*-*^ *Csf2rb2* ^*-/-*^ CD115^+^ bone marrow monocytes were transferred when compared to WT (CD45.2) monocyte transfer; interestingly, the proportion of the monocytes/macrophages and Mo-DCs recovered from the donor monocytes did not differ between the two strains (Fig. 6b), i.e., there was not a proportional loss in donor Mo-DCs in contrast to the data above wherein i.p. CAM-3003 preferentially reduced the donor monocyte to Mo-DC conversion (Fig. 5f). It can also be noted that the loss of Ly6C from the donor monocytes from WT and *Csf2rb* ^*-/-*^ *Csf2rb2* ^*-/-*^ mice was similar (Fig. 6c). The total number of CD115^+^ (R1–R3 populations) cells recovered in the recipient mice was not influenced by the strain of the monocyte donor (data not shown). Thus, direct GM-CSF signalling appears important for maintenance of cell number but not for the differentiation of monocytes into Mo-DCs, at least when certain markers are used (see “Discussion”).

## Discussion

In the AIA model we were able to show that anti-GM-CSF mAb, both prophylactically and therapeutically, could ameliorate arthritis, in particular the later structural changes. These data extend our findings with GM-CSF^-/-^ mice [25] in this model and extend the number of inflammation models where GM-CSF neutralization is effective [1, 2, 35]. In contrast, neither an anti-CSF-1R mAb nor an anti-CSF-1 mAb reduced arthritis, suggesting lack of involvement of CSF-1 and IL-34, and possibly reflecting the high neutrophil component and the relatively acute nature of the model compared with other more chronic and more macrophage-dependent arthritis models where such blockade was effective [45–47].

We took advantage of the anti-GM-CSFRα mAb (CAM-3003) to explore GM-CSFRα expression in the AIA model and the effect of GM-CSFRα blockade on synovial myeloid populations. The anti-GM-CSFRα mAb easily detected the surface GM-CSFRα on each of the F4/80^+^ MPS populations, monocytes and cDCs, but only low levels were found on neutrophils and eosinophils. GM-CSFRα blockade was successful at reducing the numbers of F4/80^+^ MPS cells and eosinophils. This observation could be clinically relevant because macrophage numbers in RA synovial tissue have been found to correlate with disease activity and it has been speculated that GM-CSF may be controlling such numbers [48]. Interestingly, among F4/80^+^ cells, Mo-DCs, defined here as MHCII^+^CD11c^+^ cells, was the only population with significantly lower numbers following anti-GM-CSFRα mAb treatment indicating a preferential reduction. Prior to the availability of CAM-3003 to measure surface murine GM-CSFRα directly and therefore easily, a chimeric protein containing the Fc-fragment of human IgG1 coupled to murine GM-CSF was the method available to measure it on myeloid populations [49].

TNF and IL-6 have been implicated in the AIA model using mAb neutralization or gene-deficient mice, albeit with variable degrees of efficacy [19–24]. Following anti-TNF or anti-IL-6 mAb treatment, unlike that of anti-GM-CSFRα mAb, we found no preferential reduction of Mo-DCs and eosinophils, but an effect on the numbers of Gr-1^+^ synovial monocytes; these data suggest that GM-CSF has a different mode of action in this model than TNF and IL-6, with possible implications for the trials targeting GM-CSF or its receptor [2]. It is possible that higher doses of anti-TNF and anti-IL-6 mAbs may have also led to a preferential reduction of Mo-DCs and eosinophils, although they were high compared to those used in other murine inflammation models [50, 51].

We also took advantage again of our convenient AIP model to explore further, using the anti-GM-CSFRα mAb approach, the role of GM-CSF in inflammation [28, 32]. This model has been viewed as a relevant, antigen-driven inflammation model to study resolution-phase macrophages given its particularly acute nature [52]. In both the AIA and AIP models, the relative surface GM-CSFRα expression of the myeloid populations was similar as was the preferential Mo-DC reduction amongst the MPS cells upon anti-GM-CSFRα administration. GM-CSFRα blockade had a significant effect on PEC numbers, whereas TNF and IL-6 depletion did not. As for murine collagen-induced arthritis [11, 33, 36], the effect on myeloid cell numbers in peritonitis upon GM-CSFRα blockade reported above was similar to that previously observed following ligand neutralization [28].

By allowing direct injection of donor monocytes into the inflamed site, the AIP model also enabled us to conclude that GM-CSF signalling in the peritoneal cavity itself was contributing to the increased numbers of inflammatory Mo-DCs and monocytes/macrophages which were shown previously to originate from infiltrating monocytes [28]. We were also able to demonstrate that GM-CSFR signalling in donor monocytes themselves regulates macrophage/Mo-DC numbers; whether this is due to a pro-survival function of GM-CSF and/or to its ability to retain cells in the cavity is unknown. In contrast, mixed bone marrow chimeras lacking functional GM-CSFR in one donor population showed that equal numbers of Mo-DCs from WT and *Csf2r* ^*-/-*^ mice were present in the inflamed central nervous system (CNS) in experimental autoimmune encephalomyelitis [3]; similarly, monocytes from WT and *Csf2r* ^*-/-*^ *Csf2rb2* ^*-/-*^ mice were able to differentiate into Mo-DCs in equal numbers in the lungs and lung-draining lymph nodes when adoptively transferred intravenously into mice infected intranasally with influenza virus [3].

Given the preferential depletion of Mo-DCs by both anti-GM-CSF [28] and anti-GM-CSFRα administration in the AIP model (Fig. 3b and c), it might have been expected that there would have been a preferential dependence for Mo-DC generation on GM-CSFR signalling in the donor monocytes. However, direct GM-CSFR signalling was not required for the differentiation of monocytes into Mo-DCs in the AIP cavity as, despite there being fewer recoverable CD115^+^ *Csf2r* ^*-/-*^ *Csf2rb2* ^*-/-*^ donor MPS cells compared to CD115^+^ WT donor MPS cells, the proportion of these donor MPS cells that had differentiated into Mo-DCs was similar between the two strains, noting that in this case the WT host cells are still able to respond to GM-CSF; the reason for this result is unclear, although it suggests an indirect GM-CSF-dependent mechanism, at least for the upregulation of the CD11c and MHCII surface markers used to define Mo-DCs.

The AIP model also enabled us to demonstrate numerous changes in gene expression between monocytes and their progeny in the inflamed peritoneal cavity. Even though the experiments above were not able to demonstrate a dramatic GM-CSF-dependence for these transcriptomic changes, it is possible that similar analyses at earlier time points may be more fruitful [52]; however, it could be that GM-CSF control over cell numbers may of itself be quite an important component of GM-CSF-dependent inflammation [27, 28, 48].

## Conclusions

In summary, we show for the first time during inflammation that GM-CSFRα neutralization leads to similar changes in myeloid populations as GM-CSF neutralization [28], with local GM-CSF signalling in MPS cells being important for the regulation of inflammatory macrophage/Mo-DC numbers. Our observations suggest that GM-CSF blockade modulates inflammatory responses differently to TNF or IL-6 blockade and may provide a greater understanding of how GM-CSFRα/GM-CSF inhibition is effective in RA; they may also help identify additional diseases where targeting this pathway may provide benefit.

## Additional files

Additional file 1: A Representative FACS plots showing Ly6G and Ly6C staining of CD45^+^ myeloid populations in the AIA knee joint. F4/80^int^SSc^hi^ eosinophils (Eos), F4/80^+^CD11c^+^MHCII^+^ Mo-DCs (R1), F4/80^+^CD11c^-^MHCII^+^ macrophages (Macs) (R2), F4/80^+^CD11c^-^MHCII^-^ macrophages (R3), F4/80^-^CD11c^+^ MHCII^+^ cDCs, F4/80^-^CD11b^+^Ly6G^+^ neutrophils, which are also Ly6C^+^, and F4/80^-^CD11b^+^Ly6G^-^SSc^lo^Ly6C^+/-^ monocytes. B Representative FACS plots of CD45^+^ myeloid populations in the AIA knee joint showing Ly6G^+^ neutrophils are CD64^-^ and F4/80^+^ macrophages/Mo-DCs are CD64^+^. (PDF 235 kb)
Additional file 2: Representative FACS plots showing the gating strategy used to identify populations in AIP. Ly6G^+^ neutrophils (Neutro), CD115^+^CD11c^+^MHCII^+^ Mo-DCs (*R1*); CD115^+^CD11c^-^MHCII^+^ macrophages (*R2*); CD115^+^CD11c^-^MHCII^-^ monocytes (*R3*), CD115^-^ Ly6G^-^CD11c^+^MHCII^+^ cDCs and CD115^-^Ly6G^-^CD11b^int^SSc^hi^ eosinophils (*Eos*). (DOCX 143 kb)
Additional file 3: Genes significantly changed in CD115^+^ cells from day 4 AIP following CAM-3003, CAT-004 or PBS treatment (day -1). CD115^+^ PECs were sorted from the peritoneal cavity of C57BL/6 on day 4 and subjected to microarray analysis. Highlighted genes were increased in CAM-3003 vs. CAT-004-treated mice; all other genes were decreased in CAM-3003- vs. CAT-004- or PBS-treated mice. (PDF 25 kb)
Additional file 4: Genes significantly changed in CD115^+^ CD45.1 donor cells from day 4 AIP. C57BL/6 CD45.1 CD115^+^ monocytes were transferred on day 2 into C57BL/6 CD45.2 recipient mice treated with CAM-3003, CAT-004 or PBS following AIP induction. CD115^+^ CD45.1 donor cells were sorted on day 4 and subjected to microarray analysis. Highlighted genes were increased in CAM-3003 vs. CAT-004-treated mice; all other genes were decreased in CAM-3003- vs. CAT-004- or PBS-treated mice. (PDF 15 kb)
Additional file 5: Array data supporting Additional file 3. Data shown as normalised intensities. (XLSX 13054 kb)
Additional file 6: Array data supporting Additional file 4. Data shown as normalised intensities. (XLSX 11003 kb)

## Abbreviations

AIA: antigen-induced arthritis
AIP: antigen-induced peritonitis
cDCs: conventional dendritic cells
CFA: complete Freund’s adjuvant
FACS: fluorescence-activated cell sorting
GM-CSF: granulocyte macrophage colony-stimulating factor
GM-CSFRα: granulocyte macrophage colony-stimulating factor receptor α − subunit
H&E: haematoxylin and eosin
i.a.: intra-articular
IL: interleukin
i.p.: intraperitoneal
KEGG: Kyoto Encyclopedia of Genes and Genomes
mAbs: monoclonal antibodies
mBSA: methylated bovine serum albumin
Mo-DCs: monocyte-derived dendritic cells
MPS: mononuclear phagocyte system
PBS: phosphate-buffered saline
PECs: peritoneal exudate cells
PG: proteoglycan
RA: rheumatoid arthritis
TNF: tumour necrosis factor
WT: wild-type
