# Supplementary figures and images for: Granulocyte macrophage colony-stimulating factor receptor α expression and its targeting in antigen-induced arthritis and inflammation
Source: Arthritis Res Ther. 2016 Dec 1;18:287. doi: 10.1186/s13075-016-1185-9 (PMC5134062; doi:10.1186/s13075-016-1185-9)

## A

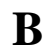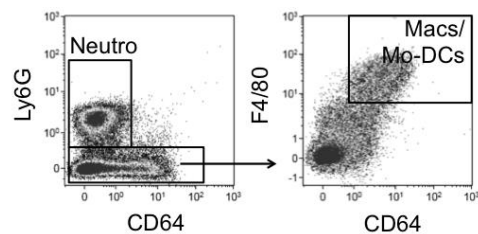

1

Supplement: Additional file 1: — A Representative FACS plots showing Ly6G and Ly6C staining of CD45+ myeloid populations in the AIA knee joint. F4/80intSSchi eosinophils (Eos), F4/80+CD11c+MHCII+ Mo-DCs (R1), F4/80+CD11c-MHCII+ macrophages (Macs) (R2), F4/80+CD11c-MHCII- macrophages (R3), F4/80-CD11c+ MHCII+ cDCs, F4/80-CD11b+Ly6G+ neutrophils, which are also Ly6C+, and F4/80-CD11b+Ly6G-SScloLy6C+/- monocytes. B Representative FACS plots of CD45+ myeloid populations in the AIA knee joint showing Ly6G+ neutrophils are CD64- and F4/80+ macrophages/Mo-DCs are CD64+. (PDF 235 kb) [file 13075_2016_1185_MOESM1_ESM.pdf]
