# Supplementary material for: Granulocyte macrophage colony-stimulating factor receptor α expression and its targeting in antigen-induced arthritis and inflammation
Source: Arthritis Res Ther. 2016 Dec 1;18:287. doi: 10.1186/s13075-016-1185-9 (PMC5134062; doi:10.1186/s13075-016-1185-9)
Supplement: Additional file 2: — Representative FACS plots showing the gating strategy used to identify populations in AIP. Ly6G+ neutrophils (Neutro), CD115+CD11c+MHCII+ Mo-DCs (R1); CD115+CD11c-MHCII+ macrophages (R2); CD115+CD11c-MHCII- monocytes (R3), CD115- Ly6G-CD11c+MHCII+ cDCs and CD115-Ly6G-CD11bintSSchi eosinophils (Eos). (DOCX 143 kb) [file 13075_2016_1185_MOESM2_ESM.docx]

**Additional file 2**


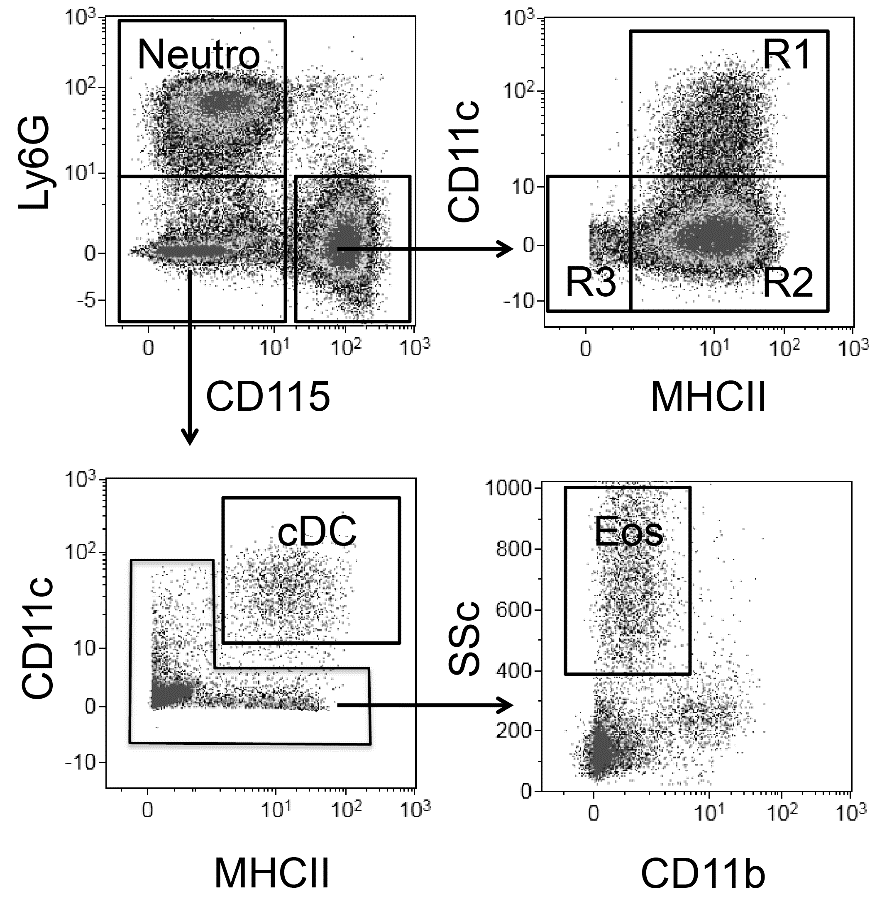


Representative FACS plots showing the gating strategy used to identify populations in AIP. Ly6G^+^ neutrophils (Neutro), CD115^+^CD11c^+^MHCII^+^ Mo-DCs (R1); CD115^+^CD11c^-^MHCII^+^ macrophages (R2); CD115^+^CD11c^-^MHCII^-^ monocytes (R3),CD115^-^ Ly6G^-^CD11c^+^MHCII^+^ cDCs and CD115^-^Ly6G^-^CD11b^int^SSc^hi^ eosinophils (Eos).
