# Supplementary material for: Granulocyte macrophage colony-stimulating factor receptor α expression and its targeting in antigen-induced arthritis and inflammation
Source: Arthritis Res Ther. 2016 Dec 1;18:287. doi: 10.1186/s13075-016-1185-9 (PMC5134062; doi:10.1186/s13075-016-1185-9)
Supplement: Additional file 3: — Genes significantly changed in CD115+ cells from day 4 AIP following CAM-3003, CAT-004 or PBS treatment (day -1). CD115+ PECs were sorted from the peritoneal cavity of C57BL/6 on day 4 and subjected to microarray analysis. Highlighted genes were increased in CAM-3003 vs. CAT-004-treated mice; all other genes were decreased in CAM-3003- vs. CAT-004- or PBS-treated mice. (PDF 25 kb) [file 13075_2016_1185_MOESM3_ESM.pdf]

Additional file 3. Genes significantly changed in CD115$^+$ cells from day 4 AIP following CAM-3003, CAT-004 or PBS treatment (day -1). CD115$^+$ PECs were sorted from the peritoneal cavity of C57BL/6 on day 4 and subjected to microarray analysis. Highlighted genes were increased in CAM-3003 vs. CAT-004-treated mice; all other genes were decreased in CAM-3003- vs. CAT-004- or PBS-treated mice.

**CAM-3003 vs. CAT-004**	
**Gene**	**Adjusted p value**
Slc36a2	6.10E-07
Gpx3	5.30E-06
Tmem154	7.00E-06
Prr15	2.90E-05
Plekhg6	0.0018
Clec4n	0.0018
Stac2	0.0019
Il1rl2	0.0024
Socs2	0.0037
Ear11	0.0079
1100001G20Rik	0.0079
Slc4a11	0.0081
**CAM-3003 vs. PBS**	
**Gene**	**Adjusted p value**
Slc36a2	2.60E-08
Il1rl2	4.40E-08

Prr15	1.40E-06
Tmem154	2.00E-06
Spint1	2.00E-06
Gpx3	2.20E-06
Ccnd1	7.70E-06
Vdr	9.70E-06
Asb4	1.00E-05
C6	1.00E-05
1100001G20Rik	1.00E-05
Stac2	2.40E-05
Clec4n	7.00E-05
Rhou	1.00E-04
Ear11	2.00E-04
Dmpk	2.00E-04
Tfec	0.00036
Slc52a3	0.00049
Sh2d1b1	0.00051
Socs2	0.00051
Rny1	0.00053
Ccl24	0.00074
Cp	0.00084
Ak4	0.00088
Nrg1	0.00091
Tgm1	0.00091
Cd28	0.001

Plekhg6	0.0011
Treml2	0.0012
Dhrs9	0.0012
Prelid2	0.0019
Cd36	0.0021
Sdc1	0.0033
Gpnmb	0.0038
Cav1	0.0044
Il1a	0.0059
Mgl2	0.0064
Vsig4	0.0065
Spp1	0.0065
Stap1	0.0068
Bcar3	0.0069
Chi3l3	0.0073
F7	0.0073
Pdcd1lg2	0.0087
Uck2	0.0089
Mmp12	0.0091
Bnip3	0.01
