# Supplementary material for: Granulocyte macrophage colony-stimulating factor receptor α expression and its targeting in antigen-induced arthritis and inflammation
Source: Arthritis Res Ther. 2016 Dec 1;18:287. doi: 10.1186/s13075-016-1185-9 (PMC5134062; doi:10.1186/s13075-016-1185-9)
Supplement: Additional file 4: — Genes significantly changed in CD115+ CD45.1 donor cells from day 4 AIP. C57BL/6 CD45.1 CD115+ monocytes were transferred on day 2 into C57BL/6 CD45.2 recipient mice treated with CAM-3003, CAT-004 or PBS following AIP induction. CD115+ CD45.1 donor cells were sorted on day 4 and subjected to microarray analysis. Highlighted genes were increased in CAM-3003 vs. CAT-004-treated mice; all other genes were decreased in CAM-3003- vs. CAT-004- or PBS-treated mice. (PDF 15 kb) [file 13075_2016_1185_MOESM4_ESM.pdf]

Additional file 4. Genes significantly changed in CD115$^+$ CD45.1 donor cells from day 4 AIP. C57BL/6 CD45.1 CD115$^+$ monocytes were transferred on day 2 into C57BL/6 CD45.2 recipient mice treated with CAM-3003, CAT-004 or PBS following AIP induction. CD115$^+$ CD45.1 donor cells were sorted on day 4 and subjected to microarray analysis. Highlighted genes were increased in CAM-3003 vs. CAT-004-treated mice; all other genes were decreased in CAM-3003- vs. CAT-004- or PBS-treated mice.

**CAM-3003 vs. CAT-004**	
**Gene**	**Adjusted p value**
Retnla	0.0009412
Ear1	0.00451
Cd36	0.008441

**CAM-3003 vs. PBS**	
**Gene**	**Adjusted p value**
F7	0.002102
Ear1	0.002102
Mrc1	0.002102
Cd36	0.002102
Retnla	0.002674
Cd24a	0.002969
Gm3601	0.002969
Prr15	0.002969
Tfec	0.002969
Ccl24	0.002969

Ednrb	0.002969
Dab2	0.002969
Tmem154	0.003402
Plxdc2	0.003626
Lrg1	0.004455
Cd200r1	0.004571
Zfp703	0.00468
Trf	0.00468
Cx3cr1	0.00468
Thbs1	0.005373
Gpx3	0.005761
Cd164	0.006773
Gpnmb	0.006912
